# Measurement Repeatability of ^18^F-FDG PET/CT Versus ^18^F-FDG PET/MRI in Solid Tumors of the Pelvis

**DOI:** 10.2967/jnumed.118.218735

**Published:** 2019-08

**Authors:** Tyler J. Fraum, Kathryn J. Fowler, John P. Crandall, Richard A. Laforest, Amber Salter, Hongyu An, Michael A. Jacobs, Perry W. Grigsby, Farrokh Dehdashti, Richard L. Wahl

**Affiliations:** 1 Mallinckrodt Institute of Radiology, Washington University School of Medicine, St. Louis, Missouri; 2 Division of Biostatistics, Washington University School of Medicine, St. Louis, Missouri; 3 Russell H. Morgan Department of Radiology and Radiological Science and Sidney Kimmel Comprehensive Cancer Center, Johns Hopkins School of Medicine, Baltimore, Maryland; 4 Department of Radiation Oncology, Washington University School of Medicine, St. Louis, Missouri; and; 5 Siteman Cancer Center, Washington University School of Medicine, St. Louis, Missouri

**Keywords:** PET/MRI, cervical cancer, repeatability, quantitative imaging, diffusion-weighted imaging, apparent diffusion coefficient

## Abstract

Knowledge of the within-subject variability of ^18^F-FDG PET/MRI measurements is necessary for proper interpretation of quantitative PET or MRI metrics in the context of therapeutic efficacy assessments with integrated PET/MRI scanners. The goal of this study was to determine the test–retest repeatability of these metrics on PET/MRI, with comparison to similar metrics acquired by PET/CT. **Methods:** This prospective study enrolled subjects with pathology-proven pelvic malignancies. Baseline imaging consisted of PET/CT immediately followed by PET/MRI, using a single 370-MBq ^18^F-FDG dose. Repeat imaging was performed within 7 d using an identical imaging protocol, with no oncologic therapy between sessions. PET imaging on both scanners consisted of a list-mode acquisition at a single pelvic station. The MRI consisted of 2-point Dixon imaging for attenuation correction, standard sequences for anatomic correlation, and diffusion-weighted imaging. PET data were statically reconstructed using various frame durations and minimizing uptake time differences between sessions. SUV metrics were extracted for both PET/CT and PET/MRI in each imaging session. Apparent diffusion coefficient (ADC) metrics were extracted for both PET/MRI sessions. **Results:** The study cohort consisted of 14 subjects (13 female, 1 male) with various pelvic cancers (11 cervical, 2 rectal, 1 endometrial). For SUV_max_, the within-subject coefficient of variation (wCV) appeared higher for PET/CT (8.5%–12.8%) than PET/MRI (6.6%–8.7%) across all PET reconstructions, though with no significant repeatability differences (all *P* values ≥ 0.08) between modalities. For lean body mass-adjusted SUV_peak_, the wCVs appeared similar for PET/CT (9.9%–11.5%) and PET/MRI (9.2%–11.3%) across all PET reconstructions, again with no significant repeatability differences (all *P* values ≥ 0.14) between modalities. For PET/MRI, the wCV for ADC_median_ of 3.5% appeared lower than the wCVs for SUV_max_ (6.6%–8.7%) and SUL_peak_ (9.2%–11.3%), though without significant repeatability differences (all *P* values ≥ 0.23). **Conclusion:** For solid tumors of the pelvis, the repeatability of the evaluated SUV and ADC metrics on ^18^F-FDG PET/MRI is both acceptably high and similar to previously published values for ^18^F-FDG PET/CT and MRI, supporting the use of ^18^F-FDG PET/MRI for quantitative oncologic treatment response assessments.

Semiquantitative assessments of ^18^F-FDG uptake on PET/CT with metrics such as the SUV are valuable tools for oncologic response assessment (1). Knowledge of measurement variability is essential to the interpretation of longitudinal changes in such metrics. Prior PET/CT studies have shown that serial measurements of ^18^F-FDG uptake are highly repeatable (2). However, ^18^F-FDG PET/CT has important limitations. For example, the low soft-tissue contrast of CT impedes primary tumor staging, whereas high background ^18^F-FDG uptake in some organs may reduce the conspicuity of metastases. Consequently, many patients also undergo MRI to improve staging accuracy.

Simultaneous PET/MRI systems can provide whole-body staging and treatment response assessment in a single examination (3). These hybrid systems have necessitated the development of MRI-based methods for attenuation correction (4). MRI-based attenuation correction maps are often affected by artifacts that can vary between imaging sessions, potentially reducing the repeatability of PET quantitation (5). In contrast, the CT-based attenuation correction approach used by PET/CT entails the direct measurement of photon attenuation by tissues, a method that is uncommonly affected by serious artifacts. The repeatability of PET metrics for PET/MRI relative to PET/CT is largely unknown.

Furthermore, PET/MRI permits the assessment of cellular density with diffusion-weighted imaging (DWI). In studies of MRI alone, quantitative metrics such as the apparent diffusion coefficient (ADC) have been used to track response to treatment or predict clinical outcomes (6,7). The repeatability of ADC metrics for integrated PET/MRI systems is unknown and might be different from MRI-only systems. For example, prior studies have suggested that the addition of PET hardware to MRI systems can worsen DWI artifacts related to eddy currents, creating the potential for greater variability in ADC values between imaging sessions (8).

Thus, the primary aim of this study was to determine the test–retest repeatability of several commonly used PET/MRI-based quantitative imaging metrics in patients with solid malignancies of the pelvis. The specific metrics of interest were SUV_max_, peak lean body mass–adjusted SUV (SUL_peak_), and ADC_median_. The repeatability of other PET and MRI metrics of potential clinical interest was also assessed in an exploratory analysis, the results of which are presented separately. A secondary aim was to evaluate the impact of various PET reconstruction techniques and frame durations on the repeatability of the above-described PET metrics.

## MATERIALS AND METHODS

### Subjects

This prospective study (ClinicalTrials.gov identifier NCT02717572) was approved by our Institutional Review Board. Inclusion and exclusion criteria are listed in Table 1. From June 2016 through May 2017, 17 subjects were enrolled. All subjects provided written informed consent. Two subjects were excluded because of failure to complete the second imaging session, and one was excluded because of lack of ^18^F-FDG uptake, which was presumably due to prior therapy.

**TABLE 1:** Inclusion and Exclusion Criteria

Parameter	Criterion
Inclusion	Histologically confirmed malignant solid tumor of pelvis (primary or metastatic; newly diagnosed or recurrent)
	Maximum tumor diameter ≥ 2.0 cm
	Age ≥ 18 y
	Ability to provide informed consent
	Ability to tolerate 60 min of supine imaging
Exclusion	Oncologic therapy within 30 d before enrollment (to minimize treatment-related changes in tumor behavior between sessions)
	Uncontrolled intercurrent illness (e.g., active infections)
	Insulin-dependent diabetes mellitus
	Prostheses incompatible with 3-T magnetic fields
	Pregnancy or nursing

### Study Protocol

**FIGURE 1.**
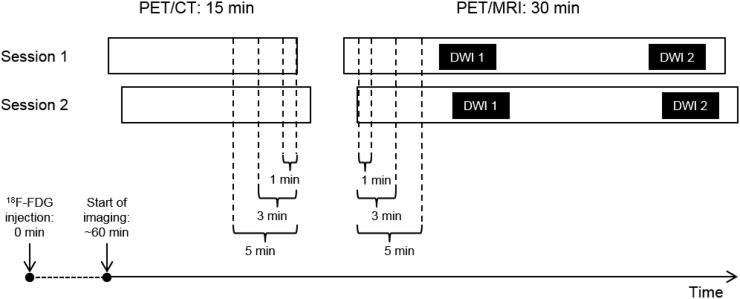
Schematic of study imaging protocol. All subjects received 370 MBq of ^18^F-FDG at 0 min. PET/CT imaging began at 60 min, lasting for 15 min. Immediately after PET/CT, PET/MRI began, lasting for 30 min. DWI was performed at beginning (DWI 1) and at end (DWI 2) of PET/MRI session. Occasionally, because of various subject-related factors, session 2 imaging began at a different time from session 1 imaging (note horizontal offset in bars representing PET/CT sessions 1 and 2 and PET/MRI sessions 1 and 2). To control for these differences, static PET images were reconstructed using overlapping intervals of 1, 3, and 5 min in PET data (brackets), thereby achieving identical effective uptake times. To minimize differences in uptake times between PET/MRI and PET/CT, these reconstruction intervals were selected from latest overlapping portion for PET/CT and earliest overlapping portion for PET/MRI.

Subjects underwent 2 imaging sessions separated by 1–7 d without any intervening oncologic treatments (Fig. 1). Immediately before the PET/MRI, subjects received 1 mg of intravenous glucagon to minimize artifacts related to bowel motility.

### PET/CT

All subjects were imaged on a Biograph 40 TruePoint/TrueView PET/CT scanner (Siemens AG). List-mode PET data were collected for 15 min for a single pelvic station (tumor centered craniocaudally within a 21.6-cm *z*-axis field of view), starting approximately 60 min after the intravenous injection of a 370-MBq ^18^F-FDG dose. A low-dose CT scan was acquired for anatomic correlation and attenuation as follows: 50 mAs (effective; CareDose tube current modulation), 120 kVp, 0.8 pitch, 0.5-s rotation time.

### PET/MRI

Immediately after PET/CT, all subjects were imaged on a Biograph mMR PET/MRI scanner, software version VA40 (Siemens AG). List-mode PET data were collected for 30 min (longer than for PET/CT because of the duration of the MRI component) for a single pelvic station (tumor centered craniocaudally within a 25.6-cm *z*-axis field of view). To minimize subject exclusion due to artifacts, DWI was performed twice during each session using the acquisition parameters in Supplemental Table 1 (supplemental materials are available at http://jnm.snmjournals.org). ADC maps were generated by the VA40 console software.

### PET Reconstructions

For each session, PET reconstructions derived from 1, 3, and 5 min of list-mode data were performed for both PET/CT and PET/MRI. For each subject, variable intervals were skipped at the beginning of each list-mode acquisition to achieve identical effective uptake times for PET/CT between sessions 1 and 2 and for PET/MRI between sessions 1 and 2 (Fig. 1). The reconstruction intervals were also selected to minimize uptake time differences between PET/CT and PET/MRI. For each frame duration, static images were generated using an ordered-subset expectation maximization reconstruction (OSEM) and an OSEM with point-spread function reconstruction (PSF), the latter of which was used to improve spatial resolution. Image reconstruction parameters are shown in Supplemental Table 2.

### Image Analysis

MIM version 6.7 (MIM Software) was used for image analysis. For each PET session, the lesion of interest was manually delineated on the basis of the perceived boundary between tumor and background, thereby generating a whole-tumor contour that was propagated to all 6 reconstructions (1 min, 3 min, 5 min; OSEM and PSF).

For each whole-tumor contour, multiple PET metrics were extracted. The term *peak* was defined as the highest mean value achievable for a 1-cm^3^ sphere placed within the lesion contour. Notably, MIM software uses the James formula for calculating the lean body mass values needed for SUL computation. For the exploratory analysis, metrics were also extracted from a 40% isocontour, which contains all voxels with an SUV at least 40% of the SUV_max_ in the whole-tumor contour, to determine the effects of semiautomated lesion segmentation on repeatability. The 40% isocontour was selected because of its correlation with metabolic tumor volume in prior PET/MRI studies of cervical cancer (9).

For each DWI acquisition, the lesion of interest was manually delineated on the corresponding ADC map, based on the perceived boundary between tumor and background diffusion properties. For each ADC contour, multiple metrics were extracted. For some acquisitions, the images were unusable because of severe artifacts related to incomplete fat suppression. For 1 subject, all 4 DWI acquisitions were inadequate. For another subject, both DWI acquisitions in the second imaging session were inadequate. Consequently, only 12 subjects could be included in the ADC repeatability analysis.

### Statistical Analysis

Subject characteristics were summarized descriptively using means ± SD for continuous metrics. The Wilcoxon signed-rank test was used to assess for differences in metric values between sessions 1 and 2.

The repeatability analysis was performed according to the methods of Bland and Altman (10). Percentage differences (%Δ) in measurements between sessions were used instead of absolute differences. For each metric, the SD of the distribution of %Δ values from all subjects was calculated. The wCV was defined as $\text{wCV }=\text{ SD}/\sqrt{2}$. The repeatability coefficient was defined as 1.96⋅SD. The %Δ between measurements is expected fall within 1 repeatability coefficient of the mean %Δ (in either direction) in approximately 95% of cases.

The normality of the %Δ%Δ distributions was assessed via visual inspection of Q-Q plots. Natural log transformation was attempted for metrics with nonnormal distributions, because repeatability statistics derived from natural log-transformed data are directly interpretable as nontransformed relative difference repeatability statistics (11). These transformations did not successfully normalize the distributions of the nonnormal metrics (data not shown). However, as stated by Bland and Altman, deviations from normality are generally not problematic in the setting of repeatability analyses, in contrast to other areas of statistics (11). Consequently, repeatability coefficients were generated from the nonnormal metrics without transformation, as denoted in the appropriate tables.

The Wilcoxon signed-rank test (paired data) or Mann–Whitney *U* test (unpaired data) was used to evaluate for significant differences in repeatability. If there are no systematic differences in measurements between sessions, the mean %Δ will be approximately 0% even when there are large %Δ values, provided that those %Δ values are randomly distributed in the positive and negative directions. In contrast, the mean |%Δ| (i.e., the mean of the absolute values of the %Δ values) will be 0% only with perfect repeatability, with larger values indicating greater magnitudes of %Δ between sessions (irrespective of whether these changes are increases or decreases). On the basis of clinical interest and relevance, a limited subset of metrics was selected for pairwise comparison via the mean |%Δ|, as the total number of possible pairs was prohibitively large.

Because of the large number of statistical tests, the methods of Benjamini and Hochberg were used to achieve a false-discovery rate of 5% (12). This correction was performed separately for the primary and exploratory aims, with statistical significance defined as *P* ≤ 0.01 and *P* ≤ 0.007, respectively. All statistical analysis was performed in R version 3.4.

## RESULTS

### Subject Characteristics

The final study cohort consisted of 14 subjects with pelvic tumors, with characteristics summarized in Table 2. All subjects but one were imaged at the time of initial cancer diagnosis. This previously treated subject had a pelvic recurrence of rectal adenocarcinoma. Mean serum glucose levels for sessions 1 and 2 were 96 and 97 mg/dL, respectively.

**TABLE 2:** Subject Characteristics

Characteristic	Data
Age (y)	48.1 ± 10.5
Height (m)	1.7 ± 0.1
Weight (kg)	82.5 ± 21.0
Body mass index (kg/m^2^)	29.7 ± 6.4
Sex	
Male	1 (7)
Female	13 (93)
Race/ethnicity	
Caucasian (non-Hispanic)	12 (86)
Caucasian (Hispanic)	1 (7)
African-American	1 (7)
Histologic diagnosis	
Cervical squamous cell carcinoma	11 (79)
Colorectal adenocarcinoma	2 (14)
Endometrial adenocarcinoma	1 (7)
Treatment status	
Initial diagnosis	13 (93)
Recurrent disease	1 (7)

Qualitative data are expressed as numbers followed by percentages in parentheses; continuous data are expressed as mean ± SD.

### PET Metrics on PET/CT Versus PET/MRI

There were systematic differences in ^18^F-FDG uptake times for PET/CT versus PET/MRI (Fig. 1). The mean endpoint for the PET/CT reconstructions was 75.1 min (range, 75–77 min) after injection. The mean start-point for the PET/MRI reconstructions was 95.8 min (range, 88–105 min) after injection.

Supplemental Table 3 shows mean values of SUV_max_ and SUL_peak_ on PET/CT versus PET/MRI for OSEM and PSF reconstructions (Supplemental Table 4 shows exploratory PET metrics). On average, the values of all PET metrics except metabolic tumor volume were numerically lower on PET/MRI than PET/CT, regardless of frame duration. These differences were statistically significant for 7 of 12 metrics (58.3%; 5 from OSEM, 2 from PSF) in the primary analysis and 12 of 72 metrics (16.7%; 11 from OSEM, 2 from PSF) in the exploratory analysis.

### ADC Metrics for PET/MRI Session 1 Versus Session 2

Supplemental Table 5 shows the mean values of ADC_median_ (Supplemental Table 6 shows exploratory ADC metrics) on PET/MRI for both imaging sessions. Mean values were nearly identical between sessions for all ADC metrics. Notably, there was no significant difference in diffusional tumor volume between sessions (*P* = 0.64).

### Repeatability of PET and ADC Metrics

**FIGURE 2.**
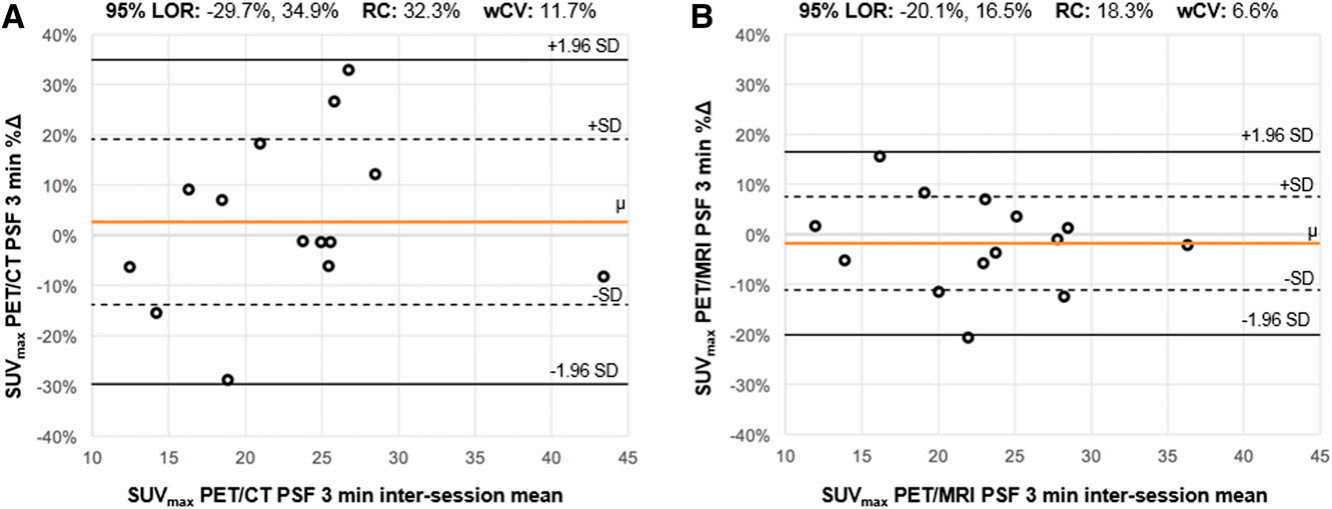
Bland–Altman plots for SUV_max_. For SUV_max_ from PSF 3-min reconstructions on PET/CT (A) and PET/MRI (B), Bland–Altman plots are shown. For each subject (**ο**), percentage change (%∆) between measurements (*y*-axis) is plotted against mean of 2 measurements. Orange line (μ) indicates mean %∆ across all subjects; dotted lines indicate 1 SD from mean %∆; solid black lines indicate 1.96 SDs from mean %∆, constituting 95% limits of repeatability (range of %∆ within which 95% of observations are expected to fall). Distance along *y*-axis between μ and either 1.96 SD line is repeatability coefficient. Comparing plots in A and B (identical *y*-axis ranges), 95% limits of repeatability appear substantially narrower for PET/MRI; correspondingly, repeatability coefficient and wCV were larger for PET/CT than PET/MRI. LOR = 95% limits of repeatability; RC = repeatability coefficient.

**FIGURE 3.**
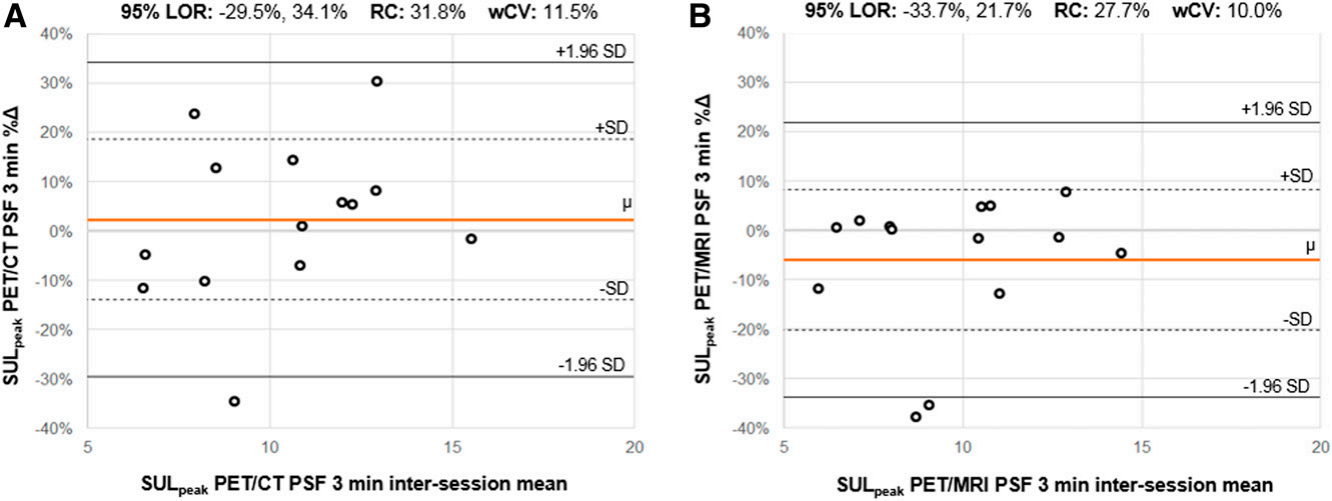
Bland–Altman plots for SUL_peak_. For SUL_peak_ from PSF 3 min reconstructions on PET/CT (A) and PET/MRI (B), Bland–Altman plots are shown. For each subject (**ο**), percentage change (%∆) between measurements (*y*-axis) is plotted against mean of 2 measurements. Orange line (μ) indicates mean %∆ across all subjects; dotted lines indicate 1 SD from mean %∆; solid black lines indicate 1.96 SDs from mean %∆, constituting 95% limits of repeatability (range of %∆ within which 95% of observations are expected to fall). Distance along *y*-axis between μ and either 1.96 SD line is repeatability coefficient. Comparing plots in A and B (identical *y*-axis ranges), 95% limits of repeatability appear slightly narrower for PET/MRI; correspondingly, repeatability coefficient and wCV were slightly larger for PET/CT than PET/MRI. LOR = 95% limits of repeatability; RC = repeatability coefficient.

**FIGURE 4.**
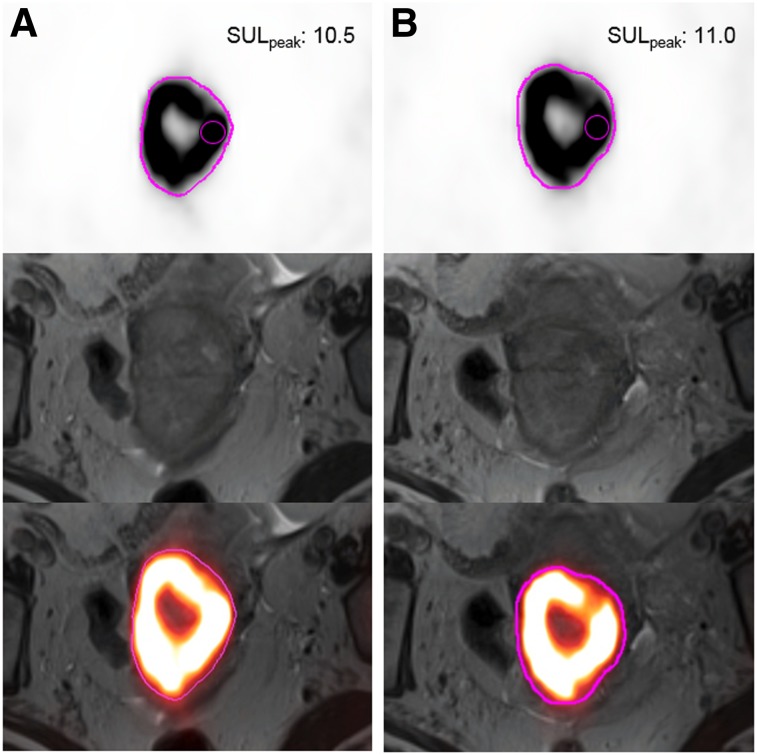
SUL_peak_ repeatability from PET image analysis. A 65-y-old woman with biopsy-proven squamous cell carcinoma of cervix was imaged with 24 h between session 1 (A) and session 2 (B). Transaxial PET images with manual whole-tumor contours (pink), T2-weighted MR images, and fused PET/MR images are shown from top to bottom for each imaging session. Note intersession contour similarities. SUL_peak_ (small pink sphere) was located in same tumor region for both sessions, with similar values.

Supplemental Table 7 shows repeatability results for SUV_max_ and SUL_peak_ on PET/CT and PET/MRI for the OSEM and PSF reconstructions (Supplemental Table 8 shows exploratory PET metrics). Relative differences between sessions, as reflected by the mean %∆ (range, −9.8%, 6.0%), were small for all metrics. For a given metric, within-subject coefficients of variation (wCVs) were generally similar across different reconstruction intervals (i.e., 1 min, 3 min, 5 min) and algorithms (i.e., OSEM, PSF). Repeatability results from the 3-min PSF reconstructions are shown in the form of Bland–Altman plots for SUV_max_ (Fig. 2) and SUL_peak_ (Fig. 3). The PET image analysis for a subject with excellent SUL_peak_ repeatability is also shown (Fig. 4).

**FIGURE 5.**
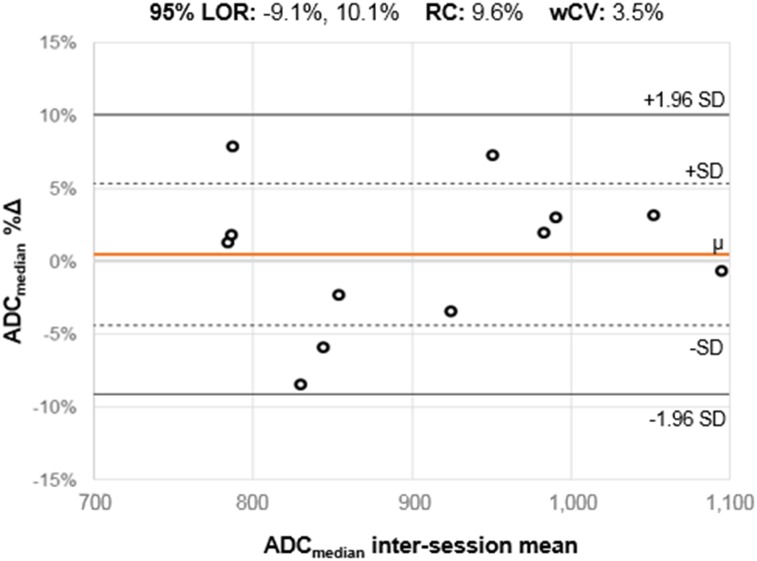
Bland–Altman plots for ADC_median_. For ADC_median_ on PET/MRI, Bland–Altman plots are shown. For each subject (**ο**), percentage change (%∆) between measurements (*y*-axis) is plotted against mean of 2 measurements. Orange line (μ) indicates mean %∆ across all subjects; dotted lines indicate 1 SD from mean %∆; solid black lines indicate 1.96 SDs from mean %∆, constituting 95% limits of repeatability (range of %∆ within which 95% of observations are expected to fall). Distance along *y*-axis between μ and either 1.96 SD line is repeatability coefficient. The 95% limits of repeatability were substantially narrower than for SUV_max_ and SUL_peak_, with numerically lower repeatability coefficient and wCV values. LOR = 95% limits of repeatability; RC = repeatability coefficient.

**FIGURE 6.**
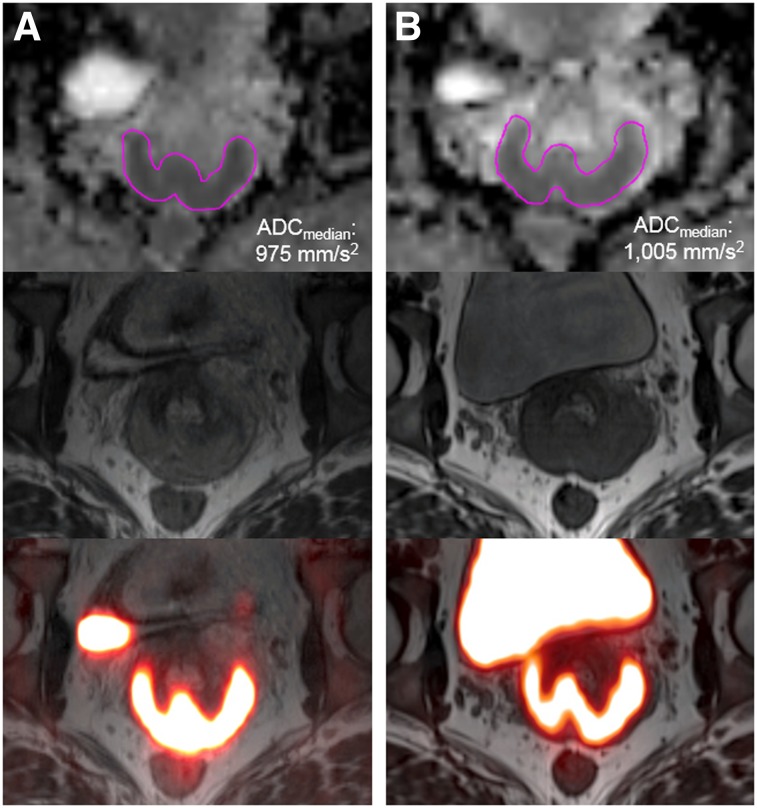
ADC_median_ repeatability from ADC map analysis. A 33-y-old woman with biopsy-proven squamous cell carcinoma of cervix was imaged with 48 h between session 1 (A) and session 2 (B). Transaxial ADC maps with manual contours (pink), T2-weighted MR images, and fused PET/MR images are shown from top to bottom for each imaging session. ADC_median_ values for sessions 1 and 2 were similar.

Supplemental Table 9 shows repeatability results for ADC_median_ on PET/MRI (Supplemental Table 10 shows exploratory ADC metrics). Relative differences between sessions, as reflected by the mean %∆ (range, −2.7%, 0.5%), were small for all metrics. Interestingly, ADC metric wCVs were generally lower than PET metric wCVs. Repeatability results for ADC_median_ are shown in the form of a Bland–Altman plot (Fig. 5). The ADC map analysis for a subject with excellent ADC_median_ repeatability is also shown (Fig. 6).

Supplemental Table 11 shows the results of pairwise repeatability comparisons of SUV_max_, SUL_peak_, and ADC_median_ (Supplemental Table 12 shows pairwise repeatability comparisons of exploratory PET and ADC metrics). Notably, for many of the comparisons of PET/CT to PET/MRI, the mean |%Δ| was numerically lower on PET/MRI, though without statistical significance. There were also no significant differences in mean |%Δ| for the PET-versus-ADC metric pairs evaluated.

## DISCUSSION

We have demonstrated the repeatability of various quantitative PET and MRI metrics on simultaneous PET/MRI. Our results point to the robustness of PET/MRI for use in clinical trials with quantitative endpoints, incorporation into treatment response assessment algorithms, and (eventually) prediction of tumor biology and clinical outcomes. Our focus on pelvic tumors reflects the expected value of PET/MRI for such malignancies based on prior studies of cervical (13–15) and rectal cancer (16). Furthermore, although other studies have assessed the repeatability of ADC metrics on MRI platforms, our study is the first (to our knowledge) to address DWI repeatability on simultaneous PET/MRI. The repeatability of DWI on PET/MRI is especially important to establish given the potential clinical value of PET/MRI-based biomarkers incorporating both SUV and ADC data (17).

With respect to the magnitude of PET metrics, values were generally lower on PET/MRI than PET/CT. These differences are likely not related to the systematically longer uptake times for PET/MRI, as delayed PET imaging generally results in higher SUVs for malignant lesions (18), but may instead be due to PET photon attenuation by the MRI body phased-array coils, which are not captured by PET/MRI attenuation correction algorithms. Furthermore, most cancers in our study arose in the cervix or rectum, both of which are surrounded by bony structures. The Siemens mMR, which uses a Dixon-based segmentation approach with 4 tissue classes (soft tissue, fat, lung, air) (4), may have undercorrected for the attenuation effects of cortical bone relative to PET/CT, resulting in lower SUVs. Newer approaches using ultrashort echo times have been successful in delineating cortical bone for attenuation correction of PET data in PET/MRI studies (19). The PSF reconstructions seemed to reduce quantitative differences between PET/MRI and PET/CT, suggesting that PSF might be best for such scanner comparisons.

With respect to the repeatability of PET metrics, our results are consistent with published results for ^18^F-FDG PET/CT. To facilitate comparisons, repeatability coefficients reported by other authors were converted as needed into wCVs. For example, a metaanalysis of 12 studies of various malignancies found a mean wCV of 11.0% for SUV_max_ (20). In our study, SUV_max_ wCVs ranged from 8.5% to 12.8% for PET/CT and 6.6% to 8.7% for PET/MRI, with similar results for the other PET metrics. Two of the studies included in the metaanalysis focused on pelvic malignancies (though neither included PET/MRI), with an SUV_max_ wCV of 10.7% for colorectal cancer (21) and 6.3% for ovarian cancer (22).

To our knowledge, there are only 2 other studies that have assessed the repeatability of PET metrics on PET/MRI. One study of head/neck cancers found SUV_max_ wCVs of 7.6% and 6.4% for PET/CT and PET/MRI, respectively, with similar wCVs for SUV_peak_ and SUV_mean_ (23). In contrast, our PET metric wCVs were generally higher, potentially reflecting differences in biology between head/neck and pelvic cancers or differences in the surrounding anatomy, as various physiologic processes specific to the pelvis (e.g., bladder filling, bowel peristalsis) might introduce greater variability between imaging sessions. As in our study, the authors found no statistically significant differences in repeatability between PET/CT and PET/MRI. The second repeatability study enrolled 33 subjects with various malignancies, including 7 colorectal cancers but no cervical cancers (24). In a single session (i.e., 1 ^18^F-FDG dose), subjects underwent either 1 PET/CT exam followed by 2 PET/MRI exams or 2 PET/MRI exams followed by 1 PET/CT exam. Despite study design differences, their PET metric wCVs for PET/MRI were generally in the 7.9%–11.2% range, on par with ours.

For the ADC metrics, our results compare favorably with those published in a recent metaanalysis of ADC repeatability for extracranial soft-tissue tumors (25). This study found a mean wCV across 12 studies of 4.1% for ADC_median_, slightly higher than our ADC_median_ wCV of 3.5%. We observed similarly low wCVs for the exploratory ADC metrics, suggesting that these metrics are also quantitatively robust. Notably, the ADC_trough_, unlike the other ADC metrics evaluated, can be determined quickly and in a relatively user-independent fashion, suggesting that it may be preferable to the other ADC measures from a clinical workflow perspective. DWI is appealing for treatment response assessment, as ADC metrics (unlike PET metrics) do not require any radiation exposure, preexamination fasting, or control for variation in uptake times between imaging sessions. However, unlike PET, DWI can be impeded by susceptibility artifacts; by chemical shift artifacts from fat suppression failure; or by intralesional fibrosis, which can develop in the tumor interstitium during treatment, resulting in low signal on ADC maps that can mimic or obscure residual or recurrent disease (26).

Our study had some limitations. First, the comparison of repeatability on PET/CT versus PET/MRI is confounded by the systematically longer uptake times for PET/MRI than for PET/CT. Although the scan order could have been randomized on a per-subject basis, this approach was not adopted because of concerns about the potential for wide ranges of uptake times within each scan type. Next, the applicability of our results to PET/MRI for other solid tumors is uncertain, as most tumors in our study were cervical cancers. It is conceivable that distinct tumor types might exhibit differences in the intrinsic variability of PET or ADC metrics. Likewise, the impact of respiratory and cardiac motion on repeatability was not tested in this study, as we focused on the pelvis only. Additionally, given that study subjects were not on any oncologic therapies between imaging sessions, it is possible (though unlikely) that there were substantial interval changes in tumor metabolism or cellularity related to true tumor growth. The number of subjects in our study was relatively small, reducing statistical power for the detection of true (though likely small) differences in repeatability between metrics. Finally, the degree to which our results, which were derived from a single model of integrated PET/MRI scanner, are generalizable to other PET/MRI scanner models is unclear, though there is no reason to suspect substantial differences.

## CONCLUSION

We have demonstrated that the test–retest measurement repeatability of various PET metrics on ^18^F-FDG PET/MRI is both acceptably high and similar to values for ^18^F-FDG PET/CT, across all PET reconstructions. These findings support the use of quantitative ^18^F-FDG PET/MRI within the framework of treatment response assessment for cervical cancer and other solid tumors of the pelvis.

## DISCLOSURE

This project was supported by NIH grants U01CA140204, 5P30CA006973 (Imaging Response Assessment Team [IRAT]), and 1R01CA190299. No other potential conflict of interest relevant to this article was reported.
