# Supplementary material for: Measurement Repeatability of 18F-FDG PET/CT Versus 18F-FDG PET/MRI in Solid Tumors of the Pelvis
Source: J Nucl Med. 2019 Aug;60(8):1080–6. doi: 10.2967/jnumed.118.218735 (PMC6681694; doi:10.2967/jnumed.118.218735)
Supplement: Supplementary file 1 [file jnm218735SupplementalData.pdf]

Supplemental Table 1. DWI acquisition parameters.

Parameter	Value
Time to repetition (TR)	5300 ms
Time to echo (TE)	81 ms
Averages	3
Field of view	330 mm x 248 mm
Voxel dimensions (in-plane)	2.6 mm x 2.6 mm
Slice thickness	5 mm
B-values	50, 500, 1000 s/mm^2
Diffusion directions	3
Fat suppression	SPAIR
Parallel acquisition technique	GRAPPA
Acceleration factor	2

Abbreviations: GRAPPA – generalized autocalibrating partial parallel acquisition; SPAIR – spectral attenuated inversion recovery

Supplemental Table 2. PET reconstruction parameters.

Parameter	OSEM		PSF	
	PET/CT	PET/MRI	PET/CT	PET/MRI
Dimensionality	2D	3D	3D	3D
Iterations	4	3	2	3
Subsets	8	21	21	21
Matrix	168 x 168	172 x 172	168 x 168	172 x 172
Zoom	1	1	1	1
Filter	5 mm Gaussian	7 mm Gaussian	2 mm Gaussian	3 mm Gaussian

Note: For the OSEM reconstructions, parameters were selected to achieve identical recovery coefficients for PET/CT and PET/MRI. Similarly, for the PSF reconstructions, manufacturer-provided resolution recovery parameters were first applied, and then the filters were adjusted to achieve identical recovery coefficients for PET/CT and PET/MRI. Time-of-flight imaging was not available for either scanner used in this study.

Abbreviations: OSEM – ordered-subset expectation maximization; PSF – point-spread function

Supplemental Table 3. Mean values of SUV_{max} and SUL_{peak} from OSEM and PSF reconstructions for PET/CT and PET/MRI.

Reconstruction	Metric	1 min			3 min			5 min		
		PET/CT	PET/MRI	*p*	PET/CT	PET/MRI	*p*	PET/CT	PET/MRI	*p*
OSEM	SUV_{max}	18.7 ± 5.6	16.3 ± 5.5	0.006*	18.3 ± 5.4	15.7 ± 5.3	0.006*	18.2 ± 5.6	16.4 ± 5.8	0.02
PSF	SUV_{max}	24.0 ± 8.0	23.6 ± 6.5	0.83	23.2 ± 7.6	22.8 ± 6.4	0.472	22.7 ± 7.7	22.0 ± 6.2	0.12
OSEM	SUL_{peak}	9.3 ± 2.5	8.2 ± 2.3	0.001*	9.3 ± 2.5	8.1 ± 2.2	0.001*	9.2 ± 2.5	8.4 ± 2.4	0.001*
PSF	SUL_{peak}	10.5 ± 2.8	9.3 ± 2.2	0.001*	10.3 ± 2.6	9.8 ± 2.5	0.036	10.3 ± 2.7	9.5 ± 2.4	0.003*

Note: All data are based on whole tumor contours. Numbers provided are means ± standard deviations across all patients and reflect values obtained for both imaging sessions averaged on a per-patient basis. The *p* values are based on results of the Wilcoxon signed-rank test.

Abbreviations: OSEM – ordered-subset expectation maximization; PSF – point spread function; SUL – lean body mass-adjusted standardized uptake value

* Significant *p* value, based on results of the Wilcoxon signed-rank test.

Supplemental Table 4. Mean values of exploratory ^{18}F-FDG-PET metrics from OSEM and PSF reconstructions for PET/CT and PET/MRI.

Reconstruction	Contour	Metric	1 min PET/CT	1 min PET/MRI	1 min *p*	3 min PET/CT	3 min PET/MRI	3 min *p*	5 min PET/CT	5 min PET/MRI	5 min *p*
OSEM	WT	SUV_{mean}	7.8 ± 2.4	7.3 ± 2.2	0.05	7.9 ± 2.3	7.1 ± 2.1	0.03	7.9 ± 2.3	7.5 ± 2.3	0.08
PSF	WT	SUV_{mean}	8.0 ± 2.3	7.3 ± 1.8	0.16	8.1 ± 2.3	8.0 ± 2.3	0.64	8.0 ± 2.3	7.8 ± 2.2	0.25
OSEM	WT	SUV_{peak}	14.8 ± 4.7	13.1 ± 4.2	0.001*	14.8 ± 4.7	12.9 ± 4.1	0.001*	14.8 ± 4.7	13.5 ± 4.4	0.001*
PSF	WT	SUV_{peak}	16.4 ± 5.0	14.9 ± 4.2	0.01	16.1 ± 4.3	15.6 ± 4.8	0.16	16.4 ± 5.2	15.3 ± 4.6	0.004*
OSEM	WT	SUV_{TLG}	611 ± 769	575 ± 778	0.25	616 ± 771	558 ± 730	0.03	615 ± 770	596 ± 790	0.33
PSF	WT	SUV_{TLG}	608 ± 759	538 ± 676	0.05	616 ± 757	615 ± 805	0.87	614 ± 755	595 ± 758	0.51
OSEM	WT	SUL_{max}	11.7 ± 2.9	10.2 ± 3.1	0.01	11.5 ± 2.8	9.8 ± 3.0	0.006*	11.4 ± 2.9	10.2 ± 3.3	0.02
PSF	WT	SUL_{max}	15.3 ± 4.2	14.7 ± 3.3	0.27	14.5 ± 4.0	14.2 ± 3.4	0.27	14.2 ± 4.0	13.7 ± 3.2	0.18
OSEM	WT	SUL_{mean}	4.9 ± 1.4	4.5 ± 1.2	0.03	5.0 ± 1.3	4.5 ± 1.1	0.006*	5.0 ± 1.3	4.7 ± 1.3	0.04
PSF	WT	SUL_{mean}	5.1 ± 1.3	4.5 ± 1.0	0.04	5.1 ± 1.3	5.0 ± 1.3	0.47	5.1 ± 1.3	4.9 ± 1.2	0.20
OSEM	WT	SUL_{TLG}	386 ± 493	361 ± 497	0.10	389 ± 495	350 ± 466	0.01	388 ± 494	374 ± 506	0.36
PSF	WT	SUL_{TLG}	391 ± 487	338 ± 431	0.01	388 ± 485	386 ± 515	0.73	387 ± 484	371 ± 483	0.20
OSEM	WT	MTV (ml)	67.8 ± 74.6	68.7 ± 77.2	0.33	67.8 ± 74.6	68.7 ± 77.2	0.33	67.8 ± 74.6	68.7 ± 77.2	0.33
PSF	WT	MTV (ml)	67.8 ± 74.6	68.7 ± 77.2	0.33	67.8 ± 74.6	68.7 ± 77.2	0.33	67.8 ± 74.6	68.7 ± 77.2	0.33
OSEM	40%	SUV_{mean}	11.0 ± 3.4	9.7 ± 3.1	0.002*	10.9 ± 3.3	9.4 ± 3.0	0.001*	10.8 ± 3.4	9.8 ± 3.2	0.02
PSF	40%	SUV_{mean}	13.4 ± 4.3	13.0 ± 3.6	0.25	13.1 ± 4.3	13.0 ± 3.9	0.60	12.9 ± 4.3	12.6 ± 3.8	0.22
OSEM	40%	SUV_{TLG}	436 ± 602	409 ± 546	0.78	463 ± 640	399 ± 495	0.18	468 ± 643	430 ± 537	0.10
PSF	40%	SUV_{TLG}	351 ± 504	266 ± 338	0.04	389 ± 546	387 ± 609	0.87	390 ± 564	390 ± 569	0.93
OSEM	40%	SUL_{mean}	6.9 ± 1.7	6.0 ± 1.7	0.002*	6.9 ± 1.7	5.9 ± 1.6	0.001*	6.8 ± 1.7	6.1 ± 1.7	0.004*
PSF	40%	SUL_{mean}	8.6 ± 2.2	8.1 ± 1.8	0.04	8.2 ± 2.2	8.1 ± 2.0	0.36	8.1 ± 2.2	7.8 ± 2.0	0.12
OSEM	40%	SUL_{TLG}	277 ± 387	257 ± 348	0.73	294 ± 412	251 ± 317	0.04	295 ± 413	271 ± 345	0.08
PSF	40%	SUL_{TLG}	226 ± 323	168 ± 216	0.004*	247 ± 350	247 ± 391	0.98	248 ± 361	245 ± 364	0.60
OSEM	40%	MTV (ml)	36.1 ± 46.8	37.6 ± 43.6	0.08	39.4 ± 51.5	38.3 ± 42.9	0.08	39.9 ± 52.2	39.4 ± 44.2	0.08
PSF	40%	MTV (ml)	25.7 ± 36.2	19.9 ± 24.8	0.01	29.6 ± 41.2	29.0 ± 44.2	0.98	29.9 ± 43.1	30.4 ± 44.0	0.83

Note: Numbers provided are means ± standard deviations across all patients and reflect values obtained for both imaging sessions averaged on a per-patient basis. SUV_{peak} and SUL_{max} were calculated only for the WT contour, as some of the 40% isocontours were not large enough for calculation of a peak value and because maximum values for the WT contour and the 40% isocontour are identical.

Abbreviations: 40% –40% isocontour; MTV – metabolic tumor volume; OSEM – ordered-subset expectation maximization; PSF – point spread function; SUL – lean body mass-adjusted standardized uptake value; TLG – total lesion glycolysis; WT –whole tumor contour

* Significant *p* value, based on results of the Wilcoxon signed-rank test.

Supplemental Table 5. Mean values of ADC_{median} from PET/MRI.

Metric	Session 1	Session 2	*p*
ADC_{median} (mm/s^2)	904 ± 104	904 ± 113	0.76

Note: Numbers provided in the session 1 and session 2 columns are means ± standard deviations across all patients. For the majority of patients, two usable DWI acquisitions were available for each imaging session. For these patients, the two data points from a given session were averaged to obtain a single value per imaging session. The *p* values are based on results of the Wilcoxon signed-rank test.

Abbreviations: ADC – apparent diffusion coefficient

Supplemental Table 6. Mean values of exploratory ADC metrics from PET/MRI.

Metric	Session 1	Session 2	*p*
ADC_{mean} (mm/s^2)	942 ± 92	933 ± 101	0.64
ADC_{20} (mm/s^2)	708 ± 79	704 ± 75	0.81
ADC_{trough} (mm/s^2)	750 ± 106	753 ± 115	0.39
DTV (ml)	64.7 ± 80.5	62.7 ± 77.5	0.64

Note: Numbers provided in the session 1 and session 2 columns are means ± standard deviations across all patients. For the majority of patients, two usable DWI acquisitions were available for each imaging session. For these patients, the two data points from a given session were averaged to obtain a single value per imaging session. The *p* values are based on results of the Wilcoxon signed-rank test.

Abbreviations: ADC – apparent diffusion coefficient; ADC_{20} – mean value obtained from the intra-lesion voxels with the lowest 20% of ADC values; ADC_{trough} – lowest mean ADC value obtainable for a 1 cm^3 sphere placed within the confines of the lesion (analogous to the inverse of *peak* for PET imaging) ; DTV – diffusional tumor volume

Supplemental Table 7. Repeatability results for SUV_{max} and SUL_{peak} from OSEM and PSF reconstructions for PET/CT and PET/MRI.

		1 min				3 min				5 min			
		PET/CT		PET/MRI		PET/CT		PET/MRI		PET/CT		PET/MRI	
Reconstruction	Metric	Mean %Δ	wCV	Mean %Δ	wCV	Mean %Δ	wCV	Mean %Δ	wCV	Mean %Δ	wCV	Mean %Δ	wCV
OSEM	SUV_{max}	-2.2%	8.5%	-5.3%	7.9%	-1.7%	9.6%	-4.7%	6.7%*	-1.6%	9.7%	-5.2%	8.0%
PSF	SUV_{max}	0.9%	12.8%	-2.3%	8.7%	2.6%	11.7%	-1.8%	6.6%	-0.8%	8.8%	-3.7%	7.7%
OSEM	SUL_{peak}	-0.6%	9.9%	-4.6%	10.4%	0.6%	10.5%	-4.7%	9.2%*	-0.2%	10.7%	-5.1%	9.7%*
PSF	SUL_{peak}	2.3%	11.2%	-3.7%	11.3%	2.3%	11.5%	-6.0%	10.0%*	0.5%	10.4%	-5.5%	9.5%*

Note: All data are based on whole tumor contours.

Abbreviations: %Δ – percent change between sessions; OSEM – ordered-subset expectation maximization; PSF – point-spread function; wCV – within-subject coefficient of variation; SUL – lean body mass-adjusted standardized uptake value

* Distribution of values for mean %Δ was non-normal.

Supplemental Table 8. Repeatability results for exploratory ^{18}F-FDG-PET parameters from OSEM and PSF reconstructions for PET/CT and PET/MRI.

			1 min				3 min				5 min			
			PET/CT		PET/MRI		PET/CT		PET/MRI		PET/CT		PET/MRI	
Reconstruction	Contour	Metric	Mean %Δ	wCV	Mean %Δ	wCV	Mean %Δ	wCV	Mean %Δ	wCV	Mean %Δ	wCV	Mean %Δ	wCV
OSEM	WT	SUV_{mean}	2.1%	12.2%	-4.2%	11.1%	3.0%	13.1%	-4.1%	10.9%	2.2%	12.9%	-4.6%	10.9%
PSF	WT	SUV_{mean}	0.1%	13.7%	-2.1%	14.4%	4.4%	14.5%	-4.8%	11.9%	2.3%	13.4%	-4.1%	11.6%
OSEM	WT	SUV_{peak}	-0.5%	9.6%	-5.8%	10.1%	0.7%	10.3%	-4.5%	8.6%*	0.7%	10.7%	-5.0%	9.4%*
PSF	WT	SUV_{peak}	-1.2%	11.2%	-3.6%	11.0%	-0.8%	14.5%	-5.9%	9.8%*	0.5%	10.1%	-5.4%	9.3%*
OSEM	WT	SUV_{TLG}	-4.2%	13.8%	-0.4%	10.5%	-3.3%	13.6%	-0.3%	10.2%	-4.0%	13.9%	-0.8%	10.4%
PSF	WT	SUV_{TLG}	-6.1%	13.9%	1.8%	12.7%	-1.9%	13.6%	-0.9%	10.2%	-4.0%	13.5%	-0.3%	9.6%
OSEM	WT	SUL_{max}	-2.3%	8.8%	-5.4%	8.2%	-1.8%	9.8%	-4.8%	7.0%*	-1.9%	10.0%	-5.3%	8.4%*
PSF	WT	SUL_{max}	4.3%	13.0%	-2.4%	8.9%	2.8%	11.8%	-1.9%	6.9%	-0.9%	9.1%	-3.8%	7.9%
OSEM	WT	SUL_{mean}	2.0%	12.5%	-4.2%	11.4%	2.9%	13.3%	-4.1%	11.1%	2.2%	13.2%	-4.7%	11.2%
PSF	WT	SUL_{mean}	3.5%	13.3%	-2.1%	14.6%	4.3%	14.7%	-4.8%	12.2%	2.2%	13.6%	-4.1%	11.9%
OSEM	WT	SUL_{TLG}	-4.2%	13.9%	-0.5%	10.8%	-3.4%	13.7%	-0.3%	10.6%	-4.1%	14.0%	-0.8%	10.7%
PSF	WT	SUL_{TLG}	-2.7%	14.0%	1.7%	12.9%	-1.7%	13.6%	-1.0%	10.5%	-4.1%	13.6%	0.3%	9.3%
OSEM	WT	MTV (ml)	-6.2%	13.8%	3.8%	9.1%*	-6.2%	13.8%	3.8%	9.1%*	-6.2%	13.8%	3.8%	9.1%*
PSF	WT	MTV (ml)	-6.2%	13.8%	3.8%	9.1%*	-6.2%	13.8%	3.8%	9.1%*	-6.2%	13.8%	3.8%	9.1%*
OSEM	40%	SUV_{mean}	-1.3%	8.2%	-5.4%	7.9%	-1.5%	9.6%	-5.4%	7.6%*	0.0%	10.4%	-5.2%	7.9%*
PSF	40%	SUV_{mean}	-0.7%	12.0%	-3.2%	8.3%	1.8%	10.5%	-4.4%	7.2%	-0.3%	9.1%	-5.4%	8.2%
OSEM	40%	SUV_{TLG}	-0.8%	16.3%	0.7%	15.0%	0.4%	13.7%	0.9%	14.1%	-2.5%	15.2%	0.7%	13.5%
PSF	40%	SUV_{TLG}	-9.8%	17.9%	-1.5%	22.2%	-0.7%	14.7%	-2.9%	18.5%	-4.5%	15.4%	0.5%	16.3%
OSEM	40%	SUL_{mean}	-1.4%	8.5%	-5.5%	8.2%	-1.6%	10.0%	-5.2%	7.4%*	-2.3%	9.8%	-5.3%	8.2%*
PSF	40%	SUL_{mean}	2.7%	11.8%	-3.3%	8.5%	1.7%	10.7%	-4.2%	7.7%	-0.4%	9.3%	-5.5%	8.5%
OSEM	40%	SUL_{TLG}	-0.9%	16.6%	0.6%	15.3%	0.4%	13.9%	0.8%	14.4%	-0.6%	14.1%	0.6%	13.8%
PSF	40%	SUL_{TLG}	-6.4%	16.5%	-1.5%	22.4%	-0.8%	14.9%	-5.1%	18.9%	-4.6%	15.6%	0.4%	16.6%
OSEM	40%	MTV (ml)	0.5%	11.9%	6.0%	14.3%*	1.9%	8.3%	6.0%	11.7%*	1.7%	9.1%	5.9%	10.6%
PSF	40%	MTV (ml)	-9.2%	15.2%	1.5%	23.0%	-2.6%	10.2%	-1.2%	17.1%	-4.2%	10.9%	5.8%	16.3%

Note: SUV_{peak} and SUL_{max} were calculated only for the WT contour, as some of the 40% isocontours were not large enough for calculation of a peak value and because maximum values for the WT contour and the 40% isocontour are identical.

Abbreviations: 40% –40% isocontour; % Δ – percent change; ^{18}F-FDG – 2-^{18}F-fluoro-2-deoxy-D-glucose; MTV – metabolic tumor volume; OSEM – ordered-subset expectation maximization; PET – positron emission tomography; PSF – point-spread function; TLG – total lesion glycolysis; wCV – within-subject coefficient of variation; WT – whole tumor contour

*Distribution of values for mean %Δ was non-normal

Supplemental Table 9. Repeatability results for ADC_{median} from PET/MRI.

	Mean %Δ	wCV
ADC_{median}	0.5%	3.5%

Note: For patients with more than one usable set of ADC data for a given session (as two DWI acquisitions were performed per session), one set was selected at random from each session for this analysis.

Abbreviations: %Δ – percent change between sessions; ADC – apparent diffusion coefficient; wCV – within-subject coefficient of variation

Supplemental Table 10. Repeatability results for exploratory ADC metrics from PET/MRI.

Metric	Mean % Δ	wCV
ADC_{mean}	-0.1%	3.4%
ADC_{20}	-0.3%	3.2%
ADC_{trough}	0.5%	3.8%
DTV	-2.7%	9.7%

Note: For patients with more than one usable set of ADC data for a given session (as two DWI acquisitions were performed per session), one set was selected at random from each session for this analysis.

Abbreviations: ADC – apparent diffusion coefficient; ADC_{20} – mean value obtained from the intra-lesion voxels with the lowest 20% of ADC values; ADC_{trough} – lowest mean ADC value obtainable for a 1 cm^3 sphere placed within the confines of the lesion (analogous to the inverse of *peak* for PET imaging) ; DTV – diffusional tumor volume; wCV – within-subject coefficient of variation

Supplemental Table 11. Pairwise comparisons of repeatability for SUV_{max}, SUL_{peak}, and ADC_{median}.

Pair	Metric 1	Mean │%Δ│	SD	Metric 2	Mean │%Δ│	SD	*p*
1	SUV_{max} OSEM 1 min PET/CT	9.4%	7.6%	SUV_{max} OSEM 1 min PET/MRI	8.0%	9.2%	0.64
2	SUL_{peak} OSEM 1 min PET/CT	11.0%	8.2%	SUL_{peak} OSEM 1 min PET/MRI	10.2%	11.2%	0.40
3	SUV_{max} OSEM 3 min PET/CT	9.9%	8.9%	SUV_{max} OSEM 3 min PET/MRI	6.5%	8.3%	0.22
4	SUL_{peak} OSEM 3 min PET/CT	10.8%	9.9%	SUL_{peak} OSEM 3 min PET/MRI	8.5%	10.7%	0.14
5	SUV_{max} OSEM 5 min PET/CT	9.7%	9.6%	SUV_{max} OSEM 5 min PET/MRI	7.7%	9.7%	0.73
6	SUL_{peak} OSEM 5 min PET/CT	10.4%	10.6%	SUL_{peak} OSEM 5 min PET/MRI	8.2%	11.9%	0.16
7	SUV_{max} PSF 1 min PET/CT	15.9%	7.6%	SUV_{max} PSF 1 min PET/MRI	9.7%	7.5%	0.08
8	SUL_{peak} PSF 1 min PET/CT	12.6%	9.3%	SUL_{peak} PSF 1 min PET/MRI	12.1%	10.4%	0.93
9	SUV_{max} PSF 3 min PET/CT	12.5%	10.5%	SUV_{max} PSF 3 min PET/MRI	7.1%	6.0%	0.12
10	SUL_{peak} PSF 3 min PET/CT	12.3%	10.4%	SUL_{peak} PSF 3 min PET/MRI	9.0%	12.3%	0.36
11	SUV_{max} PSF 5 min PET/CT	8.9%	8.5%	SUV_{max} PSF 5 min PET/MRI	8.4%	7.6%	0.87
12	SUL_{peak} PSF 5 min PET/CT	10.5%	9.9%	SUL_{peak} PSF 5 min PET/MRI	8.8%	11.4%	0.25
13	SUV_{max} PSF 3 min PET/MRI	7.1%	6.0%	ADC_{median}	3.9%	2.7%	0.23
14	SUL_{peak} PSF 3 min PET/MRI	9.0%	12.3%	ADC_{median}	3.9%	2.7%	0.74
15	SUV_{max} OSEM 3 min PET/MRI	6.5%	8.3%	ADC_{median}	3.9%	2.7%	0.90
16	SUL_{peak} OSEM 3 min PET/MRI	8.5%	10.7%	ADC_{median}	3.9%	2.7%	0.41

Note: All data are based on whole tumor contours. Numbers in the mean │%Δ│ column are means (across all subjects) of the *absolute values* of relative differences (i.e., percentage changes) between imaging sessions 1 and 2. The *p* values are based on results of the Wilcoxon signed-rank test for comparisons 1-12 (paired data) and the Mann-Whitney *U* test for comparisons 13-16 (unpaired data).

Abbreviations: ADC – apparent diffusion coefficient; OSEM – ordered-subset expectation maximization; PSF – point-spread function; SD – standard deviation

Supplemental Table 12. Pairwise comparisons of repeatability for exploratory ^{18}F-FDG-PET and ADC metrics.

Pair	Metric 1	Mean │%Δ│	SD	Metric 2	Mean │%Δ│	SD	*p*
1	Volume PET/CT	15.4%	13.0%	Volume PET/MRI	8.5%	10.1%	0.11
2	SUV_{mean} PSF 1 min PET/CT	15.6%	10.7%	SUV_{mean} PSF 1 min PET/MRI	15.2%	13.1%	0.73
3	40% SUV_{mean} PSF 1 min PET/CT	14.2%	8.4%	40% SUV_{mean} PSF 1 min PET/MRI	9.9%	6.5%	0.16
4	SUV_{mean} PSF 3 min PET/CT	16.5%	12.1%	SUV_{mean} PSF 3 min PET/MRI	12.1%	12.4%	0.18
5	40% SUV_{mean} PSF 3 min PET/CT	10.9%	9.8%	40% SUV_{mean} PSF 3 min PET/MRI	7.6%	7.9%	0.16
6	SUV_{mean} PSF 5 min PET/CT	15.1%	10.9%	SUV_{mean} PSF 5 min PET/MRI	11.8%	11.7%	0.22
7	40% SUV_{mean} PSF 5 min PET/CT	8.8%	9.0%	40% SUV_{mean} PSF 5 min PET/MRI	9.0%	8.9%	0.87
8	SUV_{mean} OSEM 1 min PET/CT	13.8%	10.0%	SUV_{mean} OSEM 1 min PET/MRI	11.3%	11.5%	0.36
9	40% SUV_{mean} OSEM 1 min PET/CT	9.0%	7.0%	40% SUV_{mean} OSEM 1 min PET/MRI	7.8%	9.4%	0.47
10	SUV_{mean} OSEM 3 min PET/CT	14.8%	10.8%	SUV_{mean} OSEM 3 min PET/MRI	11.4%	10.8%	0.25
11	40% SUV_{mean} OSEM 3 min PET/CT	9.4%	9.6%	40% SUV_{mean} OSEM 3 min PET/MRI	7.5%	9.3%	0.33
12	SUV_{mean} OSEM 5 min PET/CT	14.4%	10.7%	SUV_{mean} OSEM 5 min PET/MRI	10.3%	12.1%	0.12
13	40% SUV_{mean} OSEM 5 min PET/CT	10.3%	10.2%	40% SUV_{mean} OSEM 5 min PET/MRI	7.3%	9.8%	0.26
14	ADC_{mean} (inter-session)	3.1%	2.1%	ADC_{mean} (intra-session)	2.7%	1.9%	0.35
15	ADC_{mean} (inter-session)	3.1%	2.1%	ADC_{median} (inter-session)	3.9%	2.7%	0.90
16	ADC_{mean} (inter-session)	3.1%	2.1%	ADC_{20} (inter-session)	2.9%	3.4%	0.22
17	ADC_{mean} (inter-session)	3.1%	2.1%	ADC_{trough} (inter-session)	3.9%	3.6%	0.75
18	SUV_{max} PSF 3 min PET/MRI	7.1%	6.0%	ADC_{mean} (inter-session)	3.1%	2.1%	0.17
19	SUL_{peak} PSF 3 min PET/MRI	9.0%	12.3%	ADC_{mean} (inter-session)	3.1%	2.1%	0.70
20	SUV_{max} OSEM 3 min PET/MRI	6.5%	8.3%	ADC_{mean} (inter-session)	3.1%	2.1%	0.96
21	SUL_{peak} OSEM 3 min PET/MRI	8.5%	10.7%	ADC_{mean} (inter-session)	3.1%	2.1%	0.50
22	SUV_{mean} PSF 3 min PET/CT	16.5%	12.1%	40% SUV_{mean} PSF 3 min PET/CT	10.9%	9.8%	0.02
23	SUV_{mean} PSF 3 min PET/MRI	12.1%	12.4%	40% SUV_{mean} PSF 3 min PET/MRI	7.6%	7.9%	0.06

Note: Data are based on whole tumor contours except for metrics preceded by a '40%' designation; these metrics are based on the 40% isocontour. Numbers provided in the mean │%Δ│ column are means (across all subjects) of the *absolute values* of relative differences (i.e., percentage changes) between imaging sessions 1 and 2. The *p* values are based on results of the Wilcoxon signed-rank test for comparisons 1-17 & 22-23 (paired data) and the Mann-Whitney *U* test for comparisons 18-21 (unpaired data).

Abbreviations: 40% – 40% isocontour; ADC – apparent diffusion coefficient; OSEM – ordered-subset expectation maximization; PSF – point-spread function; WT – whole tumor
